# The pollination system of the widely distributed mammal‐pollinated *Mucuna macrocarpa* (Fabaceae) in the tropics

**DOI:** 10.1002/ece3.5201

**Published:** 2019-04-26

**Authors:** Shun Kobayashi, Tetsuo Denda, Jumlong Placksanoi, Surachit Waengsothorn, Chittima Aryuthaka, Somsak Panha, Masako Izawa

**Affiliations:** ^1^ Faculty of Science University of the Ryukyus Nishihara Japan; ^2^ Sakaerat Environmental Research Station Thailand Institute of Scientific and Technological Research Wang Nam Khieo Thailand; ^3^ Department of Marine Science Kasetsart University Bangkok Thailand; ^4^ Department of Biology Chulalongkorn University Bangkok Thailand; ^5^ Center of Excellence on Biodiversity, Ministry of Education Chulalongkorn University Bangkok Thailand

**Keywords:** explosive opening, *Mucuna macrocarpa*, non‐flying mammal, pollination, tropical Asia

## Abstract

Although the pollinators of some plant species differ across regions, only a few mammal‐pollinated plant species have regional pollinator differences in Asia. *Mucuna macrocarpa* (Fabaceae) is pollinated by squirrels, flying foxes, and macaques in subtropical and temperate islands. In this study, the pollination system of *M. macrocarpa* was identified in tropical Asia, where the genus originally diversified. This species requires “explosive opening” of the flower, where the wing petals must be pressed down and the banner petal pushed upward to fully expose the stamens and pistil. A bagging experiment showed that fruits did not develop in inflorescences (*n* = 66) with unopened flowers, whereas fruits developed in 68.7% of inflorescences (*n* = 131) with opened flowers. This indicated that the explosive opening is needed for the species to reproduce. Four potential pollinator mammals were identified by a video camera‐trap survey, and 78.3% and 60.1% of monitored inflorescences (*n* = 138) were opened by gray‐bellied squirrels (*Callosciurus caniceps*) and Finlayson's squirrels (*C. finlaysonii*), respectively, even though more than 10 mammal species visited flowers. Nectar was surrounded by the calyx, and the volume and sugar concentration of secreted nectar did not change during the day. This nectar secretion pattern is similar to those reported by previous studies in other regions. These results showed that the main pollinators of *M. macrocarpa* in the tropics are squirrels. However, the species' nectar secretion pattern is not specifically adapted to this particular pollinator. Pollinators of *M. macrocarpa* differ throughout the distribution range based on the fauna present, but there might not have been no distinctive changes in the attractive traits that accompanied these changes in pollinators.

## 1 INTRODUCTION

Plants pollinated by specific pollinators attract and limit them by specific floral traits, such as flower shape, color, and odor (Córdoba & Cocucci, 2011; Gómez et al., 2008; Hirota et al., 2012; Johnson, Burgoyne, Harder, & Dötterl, 2011). However, some widely distributed plant species with pollinator limitation seldom have the same pollinator species throughout their distribution range, because the fauna differ across the plant species' range (Boberg et al., 2014; Inoue & Amano, 1986; Johnson & Steiner, 1997; Sun, Gross, & Schiestl, 2014). In other words, plants that can be pollinated by various pollinators can increase their distribution range.

When pollinators of plants with pollinator limitation differ regionally, plants may accept alternative pollinators within the same taxon. For example, effective pollinators comprise several moth species for the orchid *Platanthera bifolia* (Boberg et al., 2014) and various bee species for *Campanula punctata* (Campanulaceae) (Nagano et al., 2014). In these examples, the pollinator species differed among the regions, but the activity time and basic shape of the pollinators were similar. On the other hand, there are some examples where the taxon and activity time of pollinators differ among regions. The pollinators of *Carnegiea gigantea* (Cactaceae) have shifted from nectar bats to birds (Fleming, Sahley, Nolland, Nason, & Hamrick, 2001). Behavior of bats differs from birds. While bats can freely use their forelimbs for feeding, birds cannot use their anatomically equivalent wings in the same manner. These shifts in pollinators are examples of regional differences in pollinators of the same plant species. Moreover, flower shape and flowering timing might also differ among regions. There are also examples of changes in the attractive traits of nectar and volatile components associated with pollinator differences (Breitkopf et al., 2013; Perret, Chautems, Spichiger, Peixoto, & Savolainen, 2001; Wester, Johnson, & Pauw, 2019). Such differences in pollination systems among different region are important to understand the speciation process. To understand the speciation process caused by shifts in pollinators, study sites should be representative of the distribution areas, although it is difficult to identify the pollination system of widely distributed plants.

**Figure 1:**
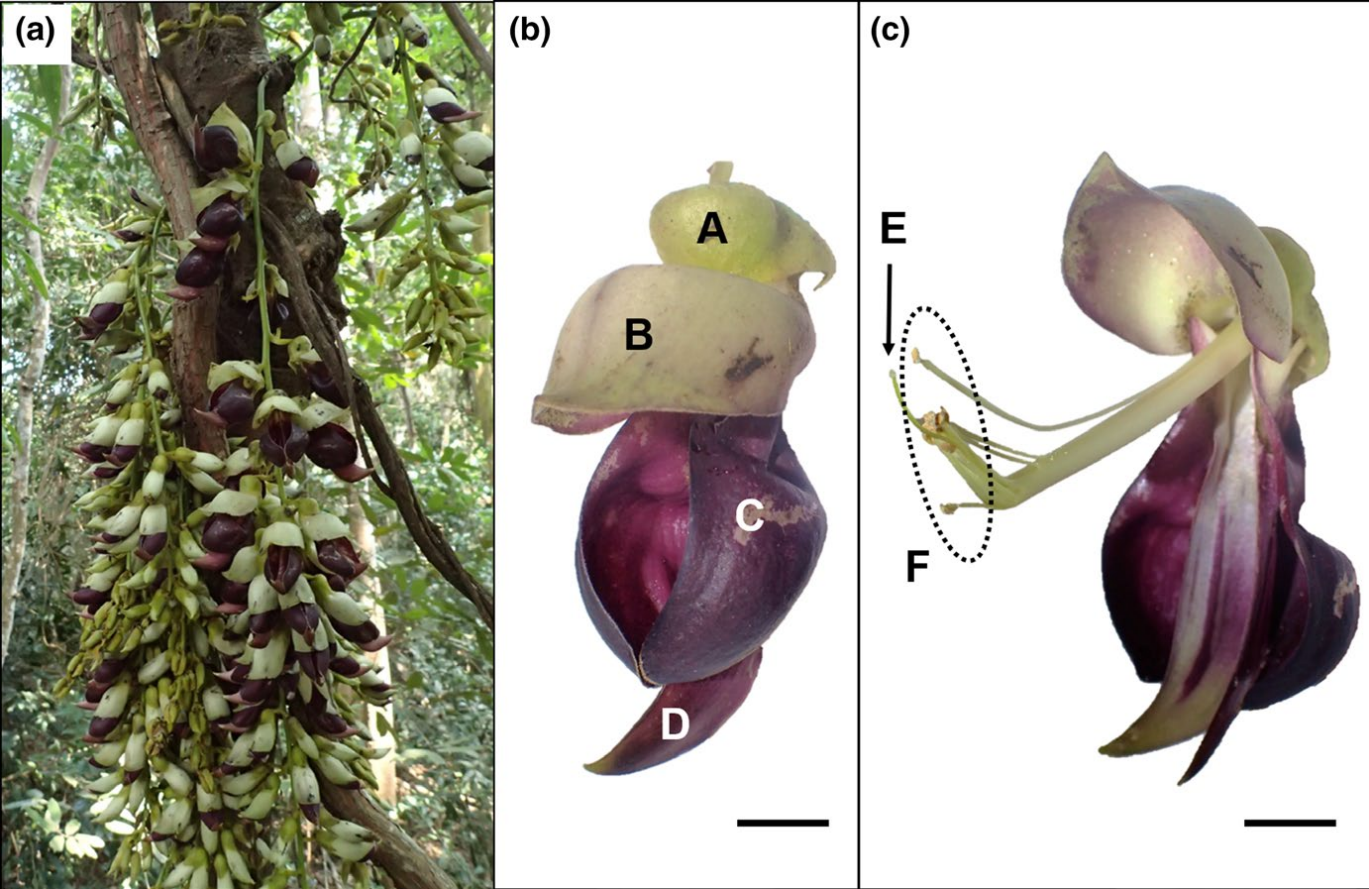
*Mucuna macrocarpa* inflorescences (a), before explosively opened flower (b), and after explosively opened flower (c). A, Calyx; B, Banner; C, Wing; D, Carina; E, Stigma; F, Anthers. Scale bars in (b) and (c) indicate 1 cm

*Mucuna macrocarpa* (Fabaceae) is a woody, evergreen, climbing vine that is widely distributed in Southeast Asia, Himalayas, Taiwan, the Ryukyu Archipelago, and Kyushu, Japan (Tateishi & Ohashi, 1981). This species shows a special “explosive opening” step during pollination (Figure 1), which is a common trait in the genus (Agostini, Sazima, & Sazima, 2006; von Helversen & von Helversen, 2003; Kobayashi, Gale, Denda, & Izawa, 2019; van der Pijl, 1941). The stamens and pistil are covered by a pair of carina petals. In *M. macrocarpa*, the banner petal must be pressed upward strongly while the wing petal must simultaneously be pushed down for the carina petals to open, thus exposing the stamens and pistil. The flower opening triggers the explosive release of a cloud of pollen grains (Kobayashi, Denda, Liao, Placksanoi, et al., 2018; Kobayashi, Hirose, Denda, & Izawa, 2018; Toyama, Kobayashi, Denda, Nakamoto, & Izawa, 2012). Once a flower explosively opens, the stamens and pistil are never covered by the carina petals. In at least two sites in Japan, this species needs explosive opening to bear fruit, because unopened flowers do not bear fruit, as experimentally in both bagged and unbagged treatments (Kobayashi, 2017). Thus, a flower‐opening animal (the “explosive opener”) is necessary to the reproduction of the plant species, making explosive openers effective pollinators.

**Figure 2:**
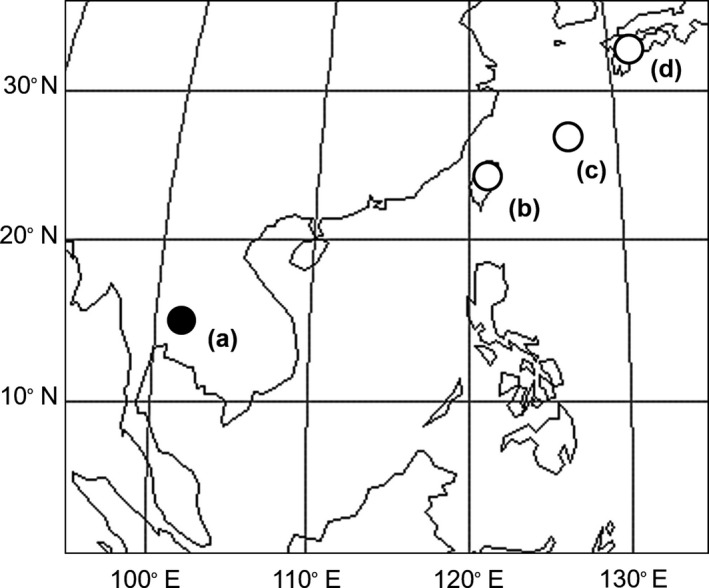
Study sites of present (a) and previous studies (b–d). (a) Sakaerat Biosphere Reserve, Nakhon Ratchasima, Thailand; (b) Taiwan; (c) Okinawa; (d) Kyushu

The explosive openers, principal pollinators, of *M. macrocarpa* are known in Kyushu, Okinawa, and Taiwan (Kobayashi, Denda, Liao, Lin, Liu, et al., 2018; Kobayashi, Denda, et al., 2017; Kobayashi et al., 2015; Toyama et al., 2012; Figure 2). The main pollinators are Japanese macaques (*Macaca fuscata*) in Kyushu (Kobayashi et al., 2015), Ryukyu flying foxes (*Pteropus dasymallus*) in Okinawa (Kobayashi, Denda, Liao, Lin, Liu, et al., 2018; Toyama et al., 2012), and red‐bellied squirrels (*Callosciurus erythraeus*) in Taiwan (Kobayashi, Denda, et al., 2017). These mammals open flowers by pushing upon the banner petal with their snout, except for Japanese macaques which open flowers using both hands, to feed on nectar (Kobayashi, Denda, Liao, Lin, Liu, et al., 2018; Kobayashi, Denda, et al., 2017; Kobayashi et al., 2015; Toyama et al., 2012). It has been suggested that sting‐like hairs on the calyx deter these animals from flower opening and prohibit nectar robbing (Toyama et al., 2012). Although there are different openers, in each region, flower shape and nectar characteristics are not clearly different among Kyushu, Okinawa, and Taiwan (Kobayashi, Denda, Liao, Lin, Wu, et al., 2018).

However, these previous studies have been conducted on subtropical and temperate islands. Because the number of mammalian species on the islands was small in almost all cases in general (Brown, 1978; Fox & Fox, 2000), the main pollinator taxon did not occur on the other islands. However, all pollinator taxa (fruit bats, macaques, and squirrels) occur sympatrically in continental tropical Southeast Asia (Duckworth, Salter, & Khounboline, 1999; Lekagul & McNeely, 1988). The aim of this study was to identify the pollination system of *M. macrocarpa* in continental tropical Southeast Asia and to compare this system among previous study sites. Accordingly, we tested the following hypotheses: (a) Pollinators of *M. macrocarpa* on islands have completely changed from the pollinator in mainland Asia, and (b) *M. macrocarpa* is pollinated by all possible mammals (bats, squirrels, and macaques) in the mainland, but differences in limited fauna present on different islands have caused pollinator changes. In addition, when several flower visitors were observed in the present study, the flower‐visiting pattern should be different among species, because Yumoto, Momose, and Nagamasu (2000) showed that the pollinators of four squirrel species visited flowers at different times. Thus, we also aimed to determine the flower‐visiting pattern of each species.

## 2 MATERIALS AND METHODS

### 2.1 Study area

This study was conducted in the dry season from February to March 2018 in the Sakaerat Biosphere Reserve (14°29ʹN, 101°52ʹE) in Nakhon Ratchasima, northeastern Thailand (Figure 2). The flowering season was January to March in the study site in this year. All possible mammalian pollinators identified by previous studies (macaques, fruit bats, squirrels, and omnivorous Carnivora) are distributed in the study area (Thailand Institute of Scientific & Technological Research, 2001). Two forest types, namely, dry deciduous forest and dry evergreen forest, are dominant in the Sakaerat Biosphere Reserve, but *M. macrocarpa* mainly grows around the edges of the latter. *Hopea ferrea* (Dipterocarpaceae) is the dominant tree species in this area, and *Shorea henryana* (Dipterocarpaceae), *Lagerstroemia duperreana* (Lythraceae), *Memecylon caeruleum* (Melastomataceae), and *Hydnocarpus ilicifolius* (Achariaceae) occur frequently (Lamotte, Gajaseni, & Malaisse, 1998).

### 2.2 Observation of flower visitors

Video camera traps (Ltl‐5210A, Ltl‐5210A940, and Ltl‐6210MC; Shenzhen Ltl Acorn Electronics Co., Ltd.) were used to monitor flower visitors. In total, 35 cameras were set up to monitor 138 inflorescences (2,198 flowers) in six plants throughout the day at various heights. Monitored plants grew within 2 ha, and three of them grew close together, while the others were at least 30 m apart. The height of inflorescences was measured to 1‐cm accuracy by a laser distance meter (Leica DISTO™ X310; Leica). When we set up the cameras, 42 flowers from 14 inflorescences had been opened by animals. Each camera was kept in place until all the flowers on a monitored inflorescence disappeared. The recording mode was set for 30‐s video clips with no interruption between clips, and sensitivity was set to normal (Kobayashi, Denda, et al., 2017; Kobayashi et al., 2015).

We calculated the flower visitation rate (VR) and explosive opening rate (EOR) of the inflorescence as shown in Equations (1) and (2):

(1) $$\text{VR}=\frac{\text{Number}\;\text{of}\;\text{inflorescences}\;\text{visited by each visitor}}{\text{Number}\;\text{of}\;\text{targeted}\;\text{inflorescences}}\times 100,\;\text{and}$$

(2) $$\text{EOR}=\frac{\text{Number}\;\text{of}\;\text{inflorescences}\;\text{with flowers opened by each visitor}}{\text{Number}\;\text{of}\;\text{targeted}\;\text{inflorescences}}\times 100.$$

The terms “VR” and “EOR” were used instead of the absolute number of flowers because flowers in an inflorescence matured individually at different times, making it difficult to determine a single flowering period using video camera traps.

The behavior of flower‐visiting animals was divided into six categories based on their effects on flowers (Kobayashi et al., 2015): (a) explosive opening with no damage to the flower (i.e., successful opening), (b) explosive opening but the flower dropped (pollen transfer may have occurred), (c) visiting an opened flower, (d) nectar robbed from an unopened flower, (e) destruction of the flower (tearing off, biting, or dropping) without opening, and (f) other behavior, such as just touching a flower. The first behavior is the only one for which a flower explosively opens and most pollen grains are removed at that time. Many pollen grains are also removed following the second behavior; however, a flower loses its female function after being picked up. The third behavior is also effective for pollination because when pollen is collected, the stigma or the pollen grains attach to the body of the animal. However, pollen grains are few at that time because most of them are removed by the explosive opener. Other behaviors do not contribute to pollination because the reproductive organs remain covered by the carina petals. In addition, once a flower was opened explosively, the direction in which the animal inserted its face into the flower was recorded as right‐side up, sideways, or upside down, since the direction of face insertion determines the position of pollen attachment (Kobayashi, Denda, et al., 2017).

### 2.3 Bagging experiments

In total, 66 inflorescences with 746 unopened flowers of four plants were covered with fine mesh nets to check the importance of the explosive opening step for fruit set and the possibility of automatic self‐pollination. Two plants grew closely, and the others were at least 30 m apart. After all flowers had dropped, fruits were counted. Fruits were also counted in the monitored flowers by video camera traps (open experiment). Fruit set rate and rate of inflorescences with fruits were then compared between bagged and open inflorescences. We counted the rate of fruit production per inflorescence for the open‐pollinated inflorescences, because we were unable to check the flower‐opening behaviors by all the explosive openers because of a time lag and otherwise missed recordings. We excluded data in which fruits in uncovered inflorescences were eaten by another animal even if a flower had been opened by an animal.

### 2.4 Nectar survey

In total, 4–6 flowers of two plants were collected every 3 hr during February 7–8 and 25–26, 2018. These plants grew individually. After the flower length was measured, the volume of nectar was measured with a microsyringe (MS‐N100; Ito Corporation), and sugar concentration (Brix index) was measured using a hand‐held refractometer (HSR‐500; Atago). Sugar composition was analyzed by high‐performance liquid chromatography (HPLC). Nectar samples used in the HPLC analysis were collected at 09:00 (*n* = 6) and 21:00 (*n* = 6) and transferred to microtubes for storage in a freezer (−20°C) until analysis. Nectar was first dissolved in acetonitrile solution (nectar:distilled water:acetonitrile = 2:33:65), which was percolated through a Mini‐UniPrep syringeless filter (UN203NPUAQU; GE Healthcare). The percolated acetonitrile solution was then analyzed by HPLC (LC‐20AD; Shimadzu Corporation). A Sugar‐D column (Nacalai Tesque) was used, and 80% acetonitrile solution was delivered at a flow rate of 0.5 ml/min. Sugars were identified from the resulting chromatogram by comparison against standard chromatograms for sucrose, glucose, and fructose, and a sugar ratio was calculated as (sucrose/[glucose + sucrose]).

Furthermore, to estimate the flower visiting and explosive opening timing of explosive openers, the width of the calyx of the opened flower and nectar volume of flowers with various calyx widths were measured. Nectar volume was subsequently estimated upon flower opening by the opener.

### 2.5 Data analysis

To examine the statistical significance of any differences, Fisher's exact test was conducted to compare the fruit set rate, the chi‐squared test to examine the height above ground level of the visit by the explosive openers, and the Mann–Whitney *U* test to compare sugar levels in the nectar. All statistical analyses were performed using R ver. 3.5.0 (R Core Team, 2018).

## 3 RESULTS

### 3.1 Flower visitors and their behavior toward flowers

**Figure 3:**
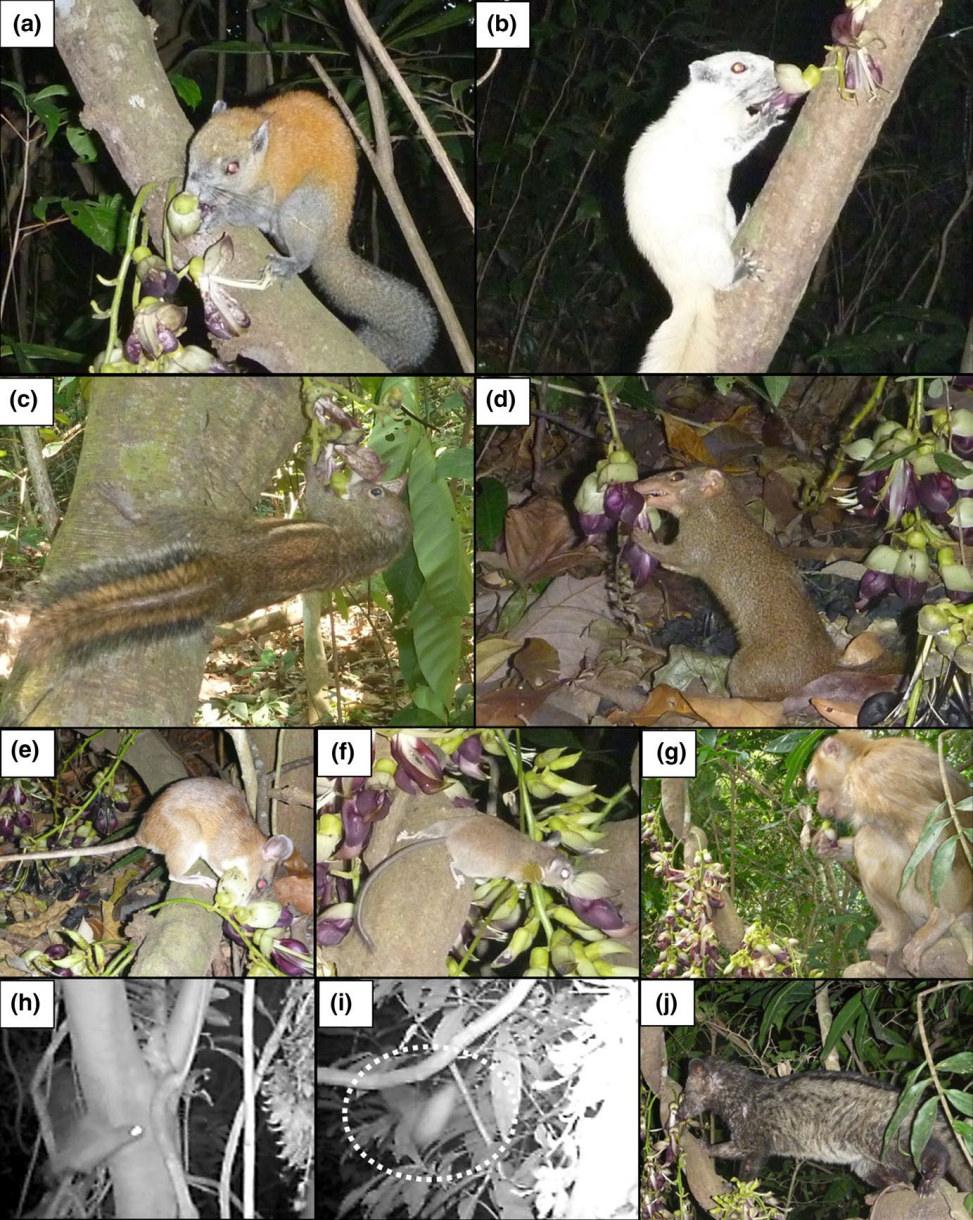
Flower visitors of *Mucuna macrocarpa* in Thailand. (a) *Callosciurus caniceps* (gray‐bellied squirrel); (b) *Callosciurus finlaysonii* (Finlayson's squirrel); (c) *Menetes berdmorei* (Indochinese ground squirrel); (d) *Tupaia glis *(common tree‐shrew); (e) *Leopoldamys sabanus *(long‐tailed giant rat); (f) Muridae species (rat); (g) *Macaca leonina* (pig‐tailed macaque); (h) Pteropodidae species (fruit bat); (i) Chiroptera species (insectivorous bat); (j) *Paradoxurus hermaphroditus* (common palm civet); a–c, and f are explosive openers

**Figure 4:**
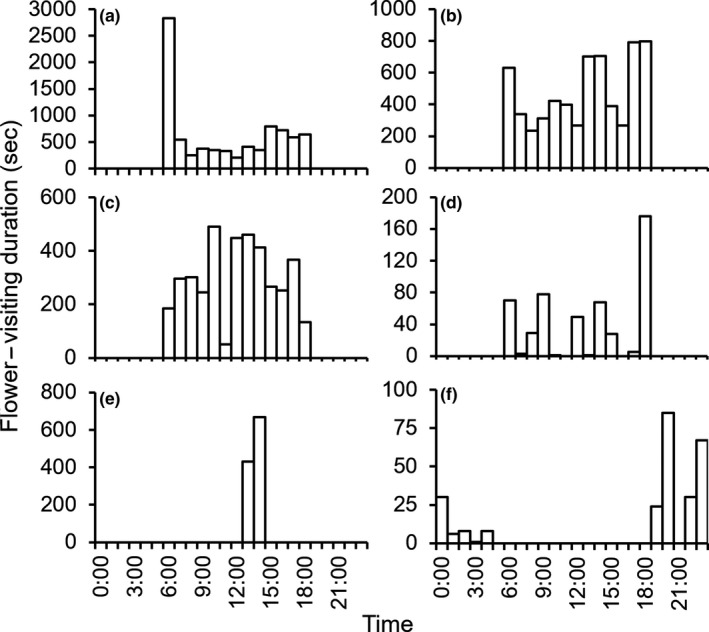
Visiting time of *Mucuna macrocarpa* flower visitors. (a) *Callosciurus caniceps* (gray‐bellied squirrel); (b) *Callosciurus finlaysonii *(Finlayson's squirrel); (c) *Menetes berdmorei* (Indochinese ground squirrel), (d) *Tupaia glis* (common tree‐shrew); (e) *Macaca leonina* (pig‐tailed macaque); (f) Muridae species (rat)

At least 10 species of mammalian flower visitors were recorded by the camera traps (Table 1; Figure 3). Gray‐bellied squirrels (*Callosciurus* *caniceps*) visited flowers most frequently (VR = 87.7%), followed by Finlayson's squirrel (*Callosciurus* *finlaysonii*) (VR = 68.1%) (Table 1). *Callosciurus caniceps* frequently visited flowers in the morning (Figure 4a), but *C*. *finlaysonii* frequently visited them in the afternoon (Figure 4b). Indochinese ground squirrels (*Menetes berdmorei*) visited flowers around noon (Figure 4c). No clear trend was detected for the other species because of their low visiting frequency. The eastern honey bee (*Apis cerana*) and several moth species also visited opened and unopened flowers.

**Table 1:** Inflorescence visitation rate (VR) by mammalian visitors to *Mucuna macrocarpa* inflorescences and explosive opening rate of inflorescences (EOR) (*n* = 138)

Flower visitors		VR (%)	EOR (%)
*Callosciurus caniceps*	Gray‐bellied squirrel	87.7	78.3
*Callosciurus finlaysonii *	Finlayson's squirrel	68.1	60.1
*Menetes berdmorei*	Indochinese ground squirrel	25.4	14.5
*Macaca leonina*	Pig‐tailed macaque	22.5	0
*Tupaia glis*	Common tree‐shrew	6.5	2.2
*Leopoldamys sabanus*	Noisy rat	5.8	0
Chiroptera sp.	Insectivorous bat sp.	4.3	0
Muridae sp.	Rat sp.	2.9	0
Pteropodidae sp.	Fruit bat sp.	0.7	0
*Paradoxurus hermaphroditus*	Common palm civet	0.7	0

Note

VR = (number of inflorescences visited by each visitor/number of monitored inflorescences) × 100. EOR = (number of inflorescences with flower opened by each visitor/number of monitored inflorescences) × 100.

**Figure 5:**
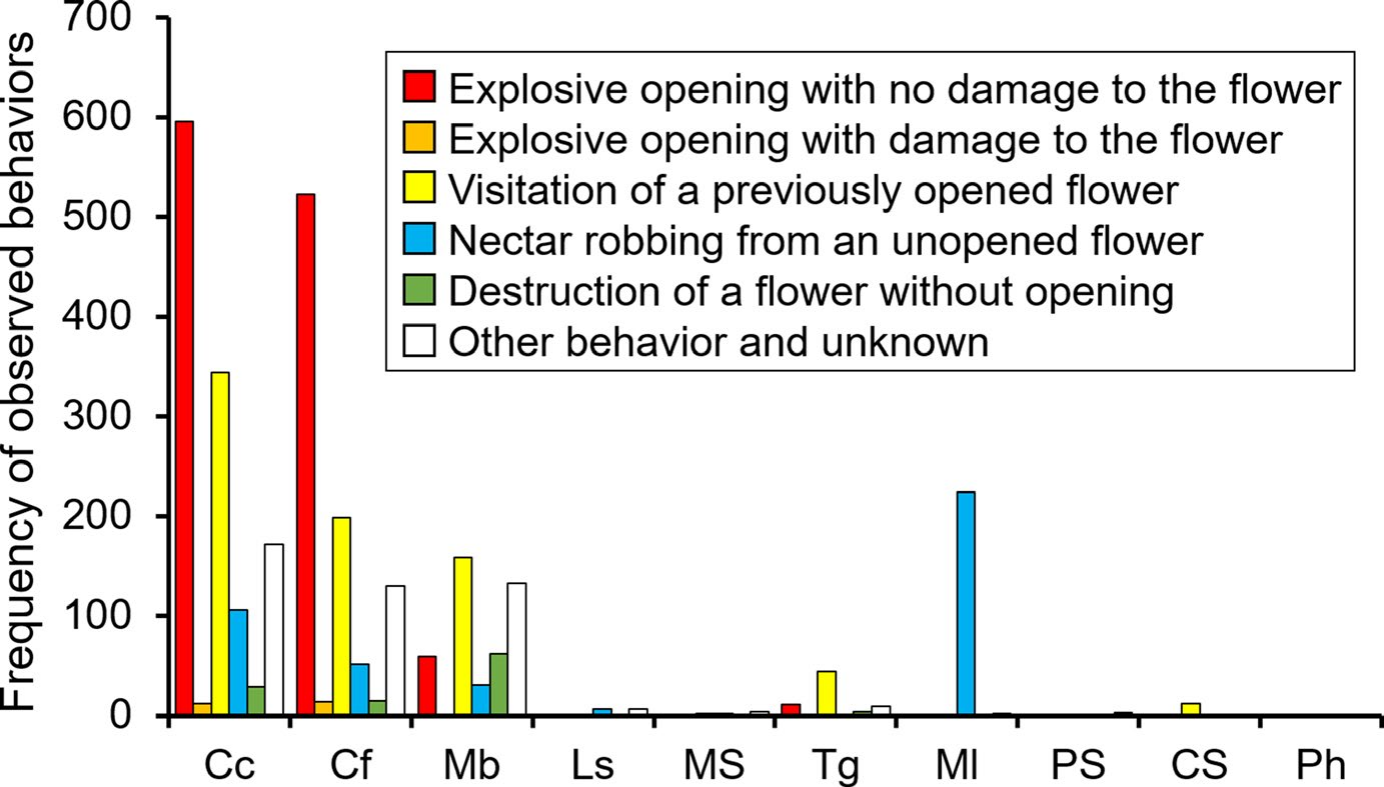
Behavior of *Mucuna macrocarpa* flower visitors. Cc, *Callosciurus caniceps* (gray‐bellied squirrel); Cf, *Callosciurus finlaysonii *(Finlayson's squirrel); CS, Chiroptera species (insectivorous bat); Ls, *Leopoldamys sabanus* (long‐tailed giant rat); Mb, *Menetes berdmorei* (Indochinese ground squirrel); Ml, *Macaca leonina* (pig‐tailed macaque); MS, Muridae species (rat); Ph, *Paradoxurus hermaphroditus* (common palm civet); PS, Pteropodidae species (fruit bat); Tg, *Tupaia glis* (common tree‐shrew)

Among the flower visitors, *C*. *caniceps*,* C*. *finlaysonii*,* M. berdmorei*, and the common tree‐shrew (*Tupaia glis*) explosively opened flowers (Table 1). The number of explosively opened flowers was highest for *C*. *caniceps* (EOR = 78.3%), and the second highest was recorded for *C*. *finlaysonii* (EOR = 60.1%) (Table 1). Although these two species frequently visited unopened flowers and explosively opened them, they also frequently visited opened flowers (Figure 5). Overall, 94.9% of monitored inflorescences were explosively opened by animals.

When visiting animals opened the flowers, they held the wing petals with a forelimb and inserted their snout into the gap between the wing and banner petals, and then pushed the banner petal upwards with their snout (right‐side up direction) (Videos S1 and S2). This behavior was common to all visitors. Most pollen grains were removed by them as evidenced by there being only a few pollen grains remaining after they had visited. The stigma made contact with the lower jaw, at the same position at which pistils adhered. *Callosciurus caniceps* opened flowers in the opposite direction in a few cases, resulting in pollen grains adhering to their head.

**Figure 6:**
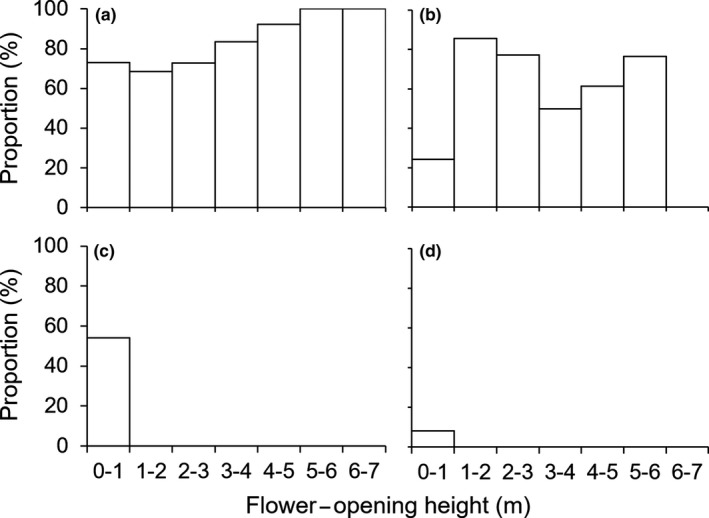
Flower‐opening height above ground level by *Mucuna macrocarpa* flower openers. (a) *Callosciurus caniceps *(gray‐bellied squirrel); (b) *Callosciurus finlaysonii *(Finlayson's squirrel); (c) *Menetes berdmorei* (Indochinese ground squirrel); (d) *Tupaia glis* (common tree‐shrew)

The height above ground level of flowers visited by the explosive openers differed among the animal species (chi‐square test; *χ* ^2^ = 77.78, *df* = 18, *p* < 0.05; Figure 6). *Callosciurus caniceps* opened flowers at various heights above ground level with a similar frequency, whereas *C. finlaysonii* opened flowers at 0–1 m at a low frequency, and *M. berdmorei* and *T. glis* opened flowers within 0–1 m above ground level.

The flower‐visiting behavior of animals other than flower openers was also recorded. Rats picked and bit flowers (Figure 5). Pig‐tailed macaques (*Macaca leonina*) were observed only on one day, when they picked flowers or inflorescences and then fed on the nectar or stamens and pistils (Figure 5).

### 3.2 Fruit set rates

No fruits were observed in the bagging experiment, and no flowers were opened when we checked the flowers dropped inside the mesh nets. In contrast, 65.2% (*n* = 138) of all monitored open inflorescences bore fruits. When we excluded the data of those cases where squirrels and macaques dropped the fruits, 71.4% (*n* = 126) of monitored inflorescences bore fruits (Table 2), and 21.2% of flowers that were opened by animals bore fruits. Fruit set rate and rate of inflorescences with fruits were significantly higher in the open experiment than in the bagging experiment (Fisher's exact test; *p* < 0.01) (Table 2). Because some inflorescences were visited by several species, we could not calculate the effect of each pollinator.

**Table 2:** Fruit set rate in the bagging experiment and monitored inflorescences and flowers in the video camera traps (open experiment)

	*n*	Rate (%)	Fisher's exact test
Bagging experiment			
Inflorescence	66	0	–
Flower	746	0	–
Open experiment			
Inflorescence			
All monitored inflorescences	138	65.2	*
Inflorescences with opened flowers	126	71.4	*
Flower			
All monitored flowers	2,160	11.6	*
Inflorescences with opened flowers	1,182	21.2	*

Note

Fisher's exact test was conducted to compare fruit set rate between bagging and open experiments. Asterisks indicate that fruit set rate was significantly higher in the open experiment than in the bagging experiment (*p* < 0.01).

### 3.3 Nectar characteristics

**Figure 7:**
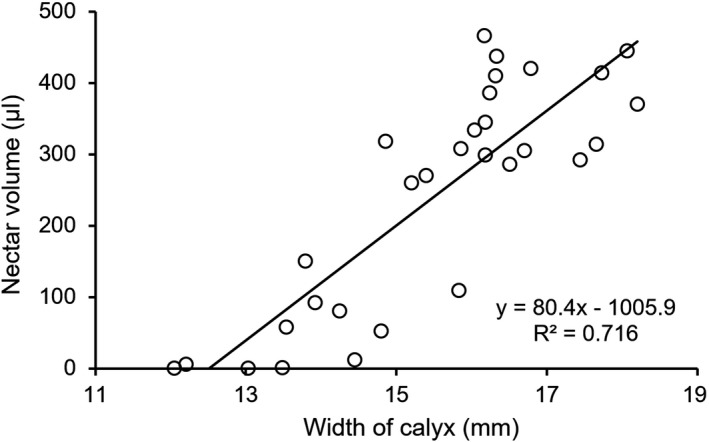
Correlation between calyx width and nectar volume of *Mucuna macrocarpa*

**Figure 8:**
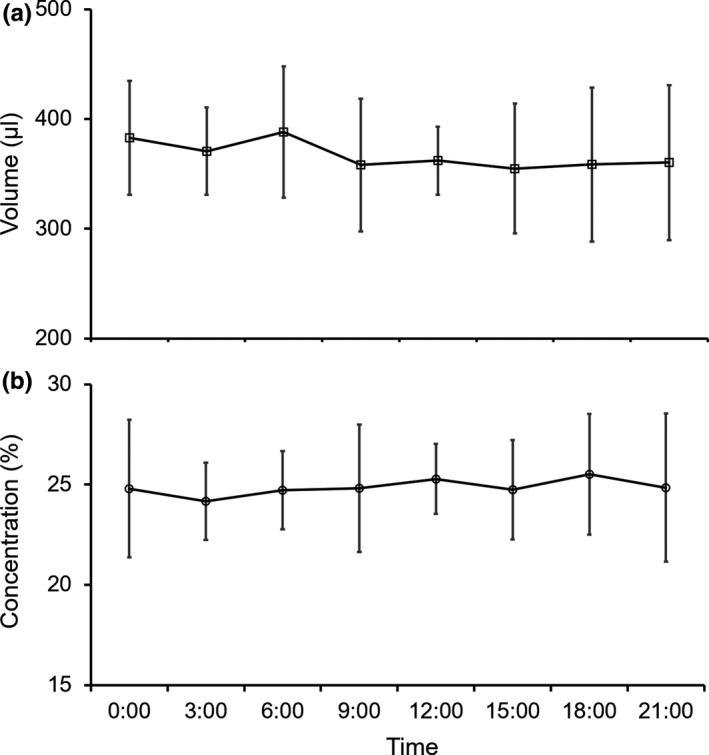
Nectar production pattern of *Mucuna macrocarpa*. (a) volume; (b) concentration

Nectar volume increased with increased calyx width (Figure 7). When a flower matured, nectar was stored throughout the day. Flower length was 65.0 ± 2.7 mm (mean ± *SD*). Nectar volume was 365.8 ± 59.5 µl (*n* = 35), and nectar concentration was 24.9 ± 2.9%; these values did not change throughout the day (Figure 8). The nectar of *M. macrocarpa* was sucrose‐dominant in both the day‐ and nighttime, with a sugar ratio of 1.34 ± 0.29 and 1.39 ± 0.34 at 09:00 and 21:00, respectively (Table 3) and no significant difference between day and night (Mann–Whitney *U* test; *W* = 16, *p* = 0.82).

**Table 3:** Sugar composition (%) and sugar ratio of *Mucuna macrocarpa* nectar. Data are shown as the mean ± *SD*

	Fructose	Glucose	Sucrose	Sugar ratio
09:00	26.1 ± 1.2	16.7 ± 4.4	57.2 ± 5.4	1.37 ± 0.29
21:00	25.4 ± 2.1	16.5 ± 4.7	8.1 ± 6.3	1.44 ± 0.34

The calyx width of flowers opened by animals was 14.13 ± 0.68 mm. The relationship between nectar volume and calyx width (Figure 7) revealed that the nectar content in flowers opened by animals did not exceed 100 µl, and these flowers were therefore opened before all the nectar was fully stored.

## 4 DISCUSSION

### 4.1 Pollination system of *M. macrocarpa* in northeastern Thailand

No fruits were observed in the bagging experiment in the present study, as reported in previous studies with similar experiments in different sites (Kobayashi, 2017). However, fruits were observed in the cross‐pollination experiment, self‐pollination experiment, and open experiment in other regions (Kobayashi, 2017). Thus, *M. macrocarpa* showed a clear requirement of explosive opening for fruit setting. In addition, explosive openers removed high quantities of pollen grains, and the stigma made contact with their lower jaw. Although some insects visited opened flowers, most pollen grains of these flowers had already been removed by the explosive openers. Therefore, the explosive openers were likely to be the effective pollinators in this study area and elsewhere (Kobayashi, Denda, Liao, Lin, Liu, et al., 2018; Kobayashi, Denda, et al., 2017; Kobayashi et al., 2015).

Explosive openers of *M. macrocarpa* in the study area included three squirrel and one tree‐shrew species, all with almost identical explosive opening behavior. In addition, many pollen grains are removed by the opener. Fruiting was recorded even when only one of these species opened a flower. Thus, the animals that opened flowers most frequently were the most effective pollinators, although we could not determine the true effectiveness because various animal species frequently visited the opened flowers and could have been pollinators. These data indicate that the most effective pollinators of *M. macrocarpa* in northeastern Thailand are likely to be *C*. *caniceps* and *C*. *finlaysonii*.

Although these two squirrel species opened flowers frequently, their interspecific relationships rendered them co‐effective pollinators. For example, four sympatric Sciuridae species (*Callosciurus prevostii*, *Sundasciurus hippurus*, *S. lowii*, and* Petaurista petaurista*) are pollinators of *Madhuca* sp., and they segregate flower‐visiting times (Yumoto et al., 2000). In the present study, the visiting time and flower height above ground level differed among the principal explosive openers, suggesting an avoidance of competition between these pollinators. Previous studies demonstrated that *C*. *caniceps* was dominant over *C*. *finlaysonii* and that *C. caniceps* visited inflorescences at higher positions than *C*. *finlaysonii* and *M. berdmorei* did (Kobayashi, Placksanoi, Taksin, Aruthaka, & Izawa, 2017; Kobayashi, Placksanoi, et al., 2019). These interspecific relationships likely enable *M. macrocarpa* to be pollinated by sympatric squirrels.

This study showed that all explosive openers visited *M. macrocarpa* flowers during the daytime. Several *Mucuna* species have become highly specialized to diurnal visitor species. For example, *M. japira*, which is pollinated by diurnal birds, stores nectar during the day, whereas the nocturnal bat‐pollinated *M. urens* blooms and secretes nectar only at night (Agostini, Sazima, & Galetto, 2011). However, *M. macrocarpa* stores nectar throughout the day in both bat‐ and squirrel‐pollinated regions, even though pollinators are reported to differ between geographic regions (Kobayashi, Denda, Liao, Lin, Wu, et al., 2018). Assuming that *M. macrocarpa* is a squirrel‐pollinated species, this nectar secretion pattern is the characteristic responsible for attracting diurnal animals, such as squirrels. As for sugar composition, a sucrose‐dominant nectar is a common feature of bat‐pollinated plants in Paleotropical regions (reviewed by Willmer, 2011). According to this review and our results, sucrose‐dominant nectar may attract not only fruit bats but also non‐flying mammals in Asia. Further studies are needed to generate data on sugar composition, especially for plants pollinated by non‐flying mammals.

### 4.2 Comparison of pollination system of *M. macrocarpa* among distribution ranges

*Mucuna macrocarpa* is pollinated by flying foxes and macaques in subtropical Okinawa and temperate Kyushu, respectively (Kobayashi, Denda, Liao, Lin, Liu, et al., 2018; Kobayashi et al., 2015; Toyama et al., 2012). Squirrels occur on neither island, flying foxes do not occur in Kyushu, and macaques are not on Okinawa (Ohdachi, Ishibashi, Iwasa, Fukui, & Saitoh, 2015). A different species of *Callosciurus* squirrel, *C. erythraeus*, is a pollinator in Taiwan, where flying foxes are absent (Kobayashi, Denda, et al., 2017). Regarding explosive opening behavior, only Japanese macaques in Kyushu, the northern limit of its range, used both hands for opening, and the others opened using their snout by holding a flower in their forelimb (Table 4). Among the main explosive openers, only flying foxes are nocturnal (Table 4). According to these results, the shift in *M. macrocarpa* pollinators may relate to the characteristics of insular fauna.

**Table 4:** Comparisons of pollinator behaviors. *F* = forelimb and SF = snout and forelimb in the explosive opening behavior column

Study region	Pollinator		Flower visiting time	Explosive opening frequency	Explosive opening behavior	Reference
Thailand	Gray‐bellied squirrel	*Callosciurus caniceps*	Day	High	SF	Present study
	Finlayson's squirrel	*Callosciurus finlaysonii*	Day	High	SF	
	Indochinese ground squirrel	*Menetes berdmorei*	Day	Low	SF	
	Common tree‐shrew	*Tupaia glis*	Day	Low	SF	
Taiwan	Red‐bellied squirrel	*Callosciurus erythraeus*	Day	High	SF	Kobayashi, Denda, et al. (2017)
	Formosan striped squirrel	*Tamiops maritimus*	Day	Low	SF	
	Masked palm civet	*Paguma larvata*	Night	Low	SF	
Okinawa	Ryukyu flying fox	*Pteropus dasymallus*	Night	High	SF	Toyama et al. (2012); Kobayashi, Denda, Liao, Lin, Liu, et al. (2018)
Kyushu	Japanese macaque	*Macaca fuscata*	Day	High	F	Kobayashi et al. (2015)
	Japanese marten	*Martes melampus*	Night	Low	SF	

Then, we estimated the pollinator shift process of *M. macrocarpa*. This genus diversified in tropical Asia and the species in the same subclade, as *M. macrocarpa* are distributed in Southeast Asia (Moura, Vatanparast, et al., 2016; Moura, Wilmot‐Dear, et al., 2016); therefore, this species might have originated in Southeast Asia. The present study revealed that squirrels are the main pollinator in Southeast Asia, indicating that the squirrel was the pollinator of *M. macrocarpa* when the plant speciated. In addition, island fauna and flora are derived from mainland fauna and flora (MacArthur & Wilson, 1967). Therefore, *M. macrocarpa* might have enlarged its distribution area from mainland Asia to the islands by changing pollinators. In this pollinator shift process, the first step was probably a shift from the mainland pollinator species of squirrels to another squirrel species within the same genus with similar flower‐opening behavior (Taiwan). The second step might have been a shift from the mainland pollinator order to a different order, but which still exhibited similar flower‐opening behavior (Okinawa). Finally, the last step would have been a shift to a pollinator different order that showed different flower‐opening behavior (Kyushu). Consequently, pollination effectiveness might also change with a shift in pollinators.

**Figure 9:**
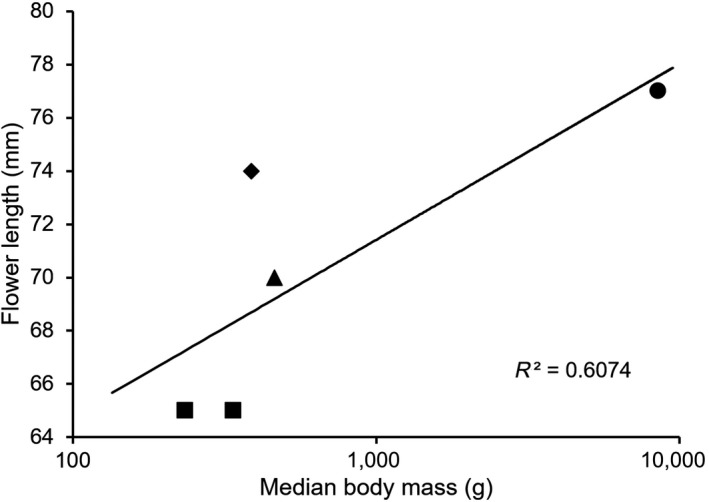
Relationships between the size of main pollinator and the flower size. Square, Squirrels (*Callosciurus caniceps* and *Callosciurus finlaysonii*) in Thailand; rhombus, squirrels (*Callosciurus erythraeus*) in Taiwan; triangle, Ryukyu flying fox (*Pteropus dasymallus*) in Okinawa; circle, Japanese macaque (*Macaca fuscata*) in Kyushu. Used body mass data of pollinator species were mean (*C. caniceps*) or median (other mammals)

When the pollinator shift is observed within a plant species, some plants adapt their floral traits to pollinators in each region (Boberg et al., 2014; Johnson & Steiner, 1997; Nagano et al., 2014; Wester et al., 2019). In *M. macrocarpa*, flowers were smaller in Thailand than in other regions (Table 5). A flower visitor must be able to engage in the explosive opening of a flower for pollination to the successful; therefore, the comparison between body size and flower size is not informative. Even so, flower size may correlate with the body mass of the main pollinator in each region (Figure 9). Conversely, nectar characteristics do not adapt to each main pollinator in each region. Nectar volume is lower in Thailand than in other regions, although it varies in all regions (Table 5). Furthermore, nectar concentration is higher in Kyushu and sugar concentration is lower in Thailand than in other regions. However, sugar composition is not different, and *M. macrocarpa* secrets sucrose‐dominant nectar in all regions (Table 5). In addition to flower shape and nectar characteristics, floral color and odor are also important for attracting mammalian pollinators (Fægri & van der Pijl, 1979; Johnson et al., 2011; Knudsen & Tollsten, 1995; Wester et al., 2019). The flowers of *M. macrocarpa* have pale green and purple petals in all regions. Pale green is one of the characteristics of plants pollinated by nocturnal animals, but purple color is frequently found in both nocturnal and diurnal animal‐pollinated plants (Willmer, 2011). Thus, we could not estimate the pollinator based on floral color. As for the odor, it emits strong smell (Toyama et al., 2012), but it is unclear whether the odor regionally differs. Therefore, further studies are needed on attractive traits. To conclude the floral traits, although external characteristics perhaps adapt to the main pollinator in each region, attractive traits such as nectar and flower color suggest that this species may attract a variety of mammals.

**Table 5:** Size of main explosive openers and floral traits of *Mucuna macrocarpa* in each region

Study region	Body mass of main explosive openers (g)		Flower length	Nectar volume	Sugar concentration	Sugar ratio	Reference
Thailand	*Callosciurus caniceps*	347 ± 25 (Male) 329 ± 26 (Female)	65.0 ± 2.7	365.8 ± 59.5	24.9 ± 2.9	1.41 ± 0.32	Present study; Saiful, Idris, Rashid, Tamura, and Hayashi (2001); Bertolino, Mazzoglio, Vaiana, and Currado (2004)
	*Callosciurus finlaysonii*	220–250					
Taiwan	*Callosciurus erythraeus*	309–457 (Male) 316–467 (Female)	73.6 ± 6.8	429.5 ± 99.2	25.4 ± 2.0	2.09 ± 0.63	Lurz, Hayssen, Geissler, and Bertolino (2013); Kobayashi, Denda, Liao, Lin, Wu, et al. (2018)
Okinawa	*Pteropus dasymallus*	393–488 (Male) 363–560 (Female)	69.7 ± 1.8	437.1 ± 86.6	24.5 ± 1.4	2.14 ± 0.44	Nakamoto, Kinjo, and Izawa (2012); Kobayashi, Denda, Liao, Lin, Wu, et al. (2018)
Kyushu	*Macaca fuscata*	7–10 kg (Female)	76.7 ± 2.3	429.4 ± 112.1	28.2 ± 1.5	1.98 ± 0.39	Kurita, Shimomura, and Fujita (2002); Kobayashi, Denda, Liao, Lin, Wu, et al. (2018)

## 5 CONCLUSION

Our results show that the main pollinators of *M. macrocarpa* in the tropics are likely to be two *Callosciurus* squirrels and that they divide visiting height and time. In other words, it is not pollinated by other possible mammals (fruit bats, macaques, and omnivorous Carnivora). Therefore, the results support our first hypothesis regarding the pollinator shift process in that the pollinators of *M. macrocarpa* on islands have completely changed from their mainland pollinator. However, although flower sizes perhaps adapt to the main pollinator in each region, attractive traits of nectar and flower color do not adapt to each pollinator in each region. Thus, flower traits of *M. macrocarpa* may not adapt to specific pollinators. Such floral traits might allow a mammal‐pollinated plants with the special pollination step expands its distribution widely.

## CONFLICT OF INTEREST

None declared.

## AUTHOR CONTRIBUTIONS

SK, TD, MI, SW, CA, and SP designed the study. All authors conducted field survey and SK mainly analyzed the data. All authors contributed to writing the paper.

## DATA AVAILABILITY STATEMENT

Raw data are available in the Dryad Digital Repository: https://doi.org/10.5061/dryad.nt71r49.
